# Spatial transcriptomics analysis identifies therapeutic targets in diffuse high-grade gliomas

**DOI:** 10.3389/fnmol.2024.1466302

**Published:** 2024-10-24

**Authors:** Yongtao Yang, Yingzhou Hong, Kai Zhao, Minhao Huang, Wenhu Li, Kui Zhang, Ninghui Zhao

**Affiliations:** ^1^Department of Neurosurgery, The Second Affiliated Hospital of Kunming Medical University, Kunming, China; ^2^Center for Life Sciences, School of Life Sciences, Yunnan University, Kunming, China

**Keywords:** glioma, isocitrate dehydrogenase, spatial transcriptomics, therapeutic target, key regulatory genes

## Abstract

**Introduction:**

Diffuse high-grade gliomas are the most common malignant adult neuroepithelial tumors in humans and a leading cause of cancer-related death worldwide. The advancement of high throughput transcriptome sequencing technology enables rapid and comprehensive acquisition of transcriptome data from target cells or tissues. This technology aids researchers in understanding and identifying critical therapeutic targets for the prognosis and treatment of diffuse high-grade glioma.

**Methods:**

Spatial transcriptomics was conducted on two cases of isocitrate dehydrogenase (IDH) wild-type diffuse high-grade glioma (Glio-IDH-wt) and two cases of IDH-mutant diffuse high-grade glioma (Glio-IDH-mut). Gene set enrichment analysis and clustering analysis were employed to pinpoint differentially expressed genes (DEGs) involved in the progression of diffuse high-grade gliomas. The spatial distribution of DEGs in the spatially defined regions of human glioma tissues was overlaid in the t-distributed stochastic neighbor embedding (t-SNE) plots.

**Results:**

We identified a total of 10,693 DEGs, with 5,677 upregulated and 5,016 downregulated, in spatially defined regions of diffuse high-grade gliomas. Specifically, *SPP1*, *IGFBP2*, *CALD1*, and *TMSB4X* exhibited high expression in carcinoma regions of both Glio-IDH-wt and Glio-IDH-mut, and 3 upregulated DEGs (*SMOC1*, *APOE*, and *HIPK2*) and 4 upregulated DEGs (*PPP1CB*, *UBA52*, *S100A6*, and *CTSB*) were only identified in tumor regions of Glio-IDH-wt and Glio-IDH-mut, respectively. Moreover, Kyoto Encyclopedia of Genes and Genomes (KEGG) and gene ontology (GO) enrichment analyses revealed that upregulated DEGs were closely related to PI3K/Akt signaling pathway, virus infection, and cytokine-cytokine receptor interaction. Importantly, the expression of these DEGs was validated using GEPIA databases. Furthermore, the study identified spatial expression patterns of key regulatory genes, including those involved in protein post-translational modification and RNA binding protein-encoding genes, with spatially defined regions of diffuse high-grade glioma.

**Discussion:**

Spatial transcriptome analysis is one of the breakthroughs in the field of medical biotechnology as this can map the analytes such as RNA information in their physical location in tissue sections. Our findings illuminate previously unexplored spatial expression profiles of key biomarkers in diffuse high-grade glioma, offering novel insight for the development of therapeutic strategies in glioma.

## 1 Introduction

Diffuse high-grade gliomas are among the most aggressive primary brain tumors, characterized by invasive growth, rapid progress, incomplete resection challenges, frequent recurrence, and poor prognosis (Jo et al., 2023). Intratumoral heterogeneity poses the greatest obstacle in treating these tumors (Comba et al., 2021), influencing clinical presentation, treatment efficacy, sensitivity variations, and drug resistance. While multimodal treatment strategies, including surgical resection, radiotherapy, chemotherapy, targeted drug therapy, and supportive care (Luo et al., 2023), have improved patient prognosis, the median survival time remains a bleak 14.6 months (Zou et al., 2013). Therefore, identifying effective therapeutic targets is crucial to enhance patient outcomes.

Recent advancements in sequencing technologies have revolutionized tumor research (Nakagawa and Fujita, 2018). Biomarker studies, evolving from single-omics to multi-omics integration (encompassing genomics, transcriptomics, epigenetics, microbiome, metabolomics, proteomics, and radiomics), have enhanced diagnosis and prognostic capabilities across various cancers (Qi et al., 2020). Multi-omics approaches have revealed the immune response mechanisms in gliomas (Cheng et al., 2021; Johnson et al., 2022), uncovering novel biomarkers for treatment response prediction and prognosis. High-throughput sequencing of gene expression profiles aids personalized treatment strategies (Goodwin et al., 2016), yes lack spatial cellular information crucial for understanding cell differentiation and interactions during tumorigenesis (Ståhl et al., 2016).

Spatial transcriptome sequencing, an emerging technique, integrates gene expression with cellular spatial information, offering new insight into tumor heterogeneity, microenvironment, and immunity of tumors (Du et al., 2023; Wang X. et al., 2023). For example, Berglund et al. (2018) highlighted NR4A1 as highly expressed in the reactive stroma of prostate cancer sections. Wang F. et al. (2023), demonstrated the enrichment of MCAM^+^ fibroblasts in liver metastatic tumors using scRNA-seq combined with spatial transcriptomics, revealing their role in promoting CD8_CXCL13 cell generation via the Notch pathway, potentially accelerating liver metastatic colorectal cancer development. Spatial transcriptome sequencing also annotates tissue components, unveiling previously unexplored heterogeneity landscapes. Moreover, recent work integrating scRNA-seq with spatial transcriptomics across 16 glioblastomas confirmed chemotactic attraction of cancer-associated fibroblasts (CAFs) toward glioblastoma stem cells (GSCs), wherein CAFs enriched GSCs via upregulation of osteopontin and hepatocyte growth factor (Jain et al., 2023). Similarly, another study showed that spatial transcriptomics revealed niche-specific enrichment and vulnerabilities of radial glial stem-like cells in malignant gliomas (Ren et al., 2023). Zhao et al. (2023) reported that combining scRNA-seq and spatial transcriptome identified BARD1 as a potential therapeutic target for glioblastoma patients. Of note, spatial transcriptomics can not only present an overall view of the immune microenvironment in glioma, but also be used to explore the dynamic development and spatial specificity of immune cells in the microenvironment (Buehler et al., 2023; Sun et al., 2023). Therefore, spatial transcriptome technology may help us to capture genome-wide readouts across biological tissue space and identify therapeutic biomarkers that may have prognostic significance in glioma.

In this study, we utilize spatial transcriptome sequencing to delineate comprehensive spatial characteristics of diffuse high-grade glioma. Specifically, we identify potential therapeutic targets from spatially defined regions of IDH wild-type and mutant high-grade glioma, validating these targets through public databases. Additionally, our analysis uncovers key regulatory genes in ubiquitination, RNA binding protein, and kinase influencing the spatial biology of high-grade glioma. These findings provide potential targets for prognosis and precision therapy in glioma management.

## 2 Materials and methods

### 2.1 Sample information

Two formalin-fixed paraffin-embedded (FFPE) tissues of patients with IDH-wild-type diffuse high-grade glioma (Glio-IDH-wt) and two FFPE tissues of patients with IDH-mutant diffuse high-grade glioma (Glio-IDH-mut) were collected for further spatial transcriptome RNA sequencing. This study involving human FFPE samples was reviewed and approved by The Second Affiliated Hospital of Kunming Medical University (No: APPROVED-PJ-SCIENTIFIC-2024-71).

### 2.2 Spatial sequencing library preparation (FFPE-V2)

Formalin-fixed, paraffin-embedded (FFPE) samples passing the RNA quality control (DV200 > 30%) were used for spatial transcriptomic construction and sequencing. 5-micron thick sections were mounted onto a Visium Gene Expression slide (10X Genomics), baked at 42°C for 3 h, and dried in a desiccator at room temperature overnight. For deparaffinization, the slide was incubated at 60°C for 2 h, immersed in xylene, and rehydrated in an ethanol gradient. Hematoxylin–eosin staining (H&E) staining was then performed using Mayer’s hematoxylin (Millipore Sigma), bluing reagent (Dako, Agilent), and alcoholic eosin (Millipore Sigma). Stained slides were scanned under a microscope, followed by decrosslinking using 0.1 N HCl and TE Buffer (pH 9.0) to release RNA that was sequestered by formalin. The stained slide was incubated with a Human whole transcriptome probe panel and then transferred to Cytassist (10X Genomics).

A human whole transcriptome probe panel (10X) that consisted of three pairs of specific probes (5′ containing Small RNA Read 2S and 3′ containing poly-A) for mostly genes was hybridized to RNA. Probe pairs were then ligated to seal the junctions between them and to form the single-stranded ligation products. The samples were treated with RNase and permeabilized to release the ligation products. A poly-A portion of the products was then captured by the poly (dT) regions of the capture probes percolated on the Visium slide that also includes an Illumina Read 1, spatial. Barcode, and unique molecular identifier (UMI). Probes were extended to produce spatially barcoded ligated probe products and released from the slide for indexing via Sample Index PCR and final library construction and sequencing. Visium Spatial Gene Expression libraries consisted of Illuminapaired-end sequences flanked with P5/P7. The 16-bp Spatial Barcode and 12-bp UMI were encoded in Read 1, while Read 2S was used to sequence the ligated probe insert.

### 2.3 Sequencing data processing

After sequencing, raw fastq files, and bright field H&E images are processed by Space Ranger (version 2.1.0) to align to genome GRCh38 (downloaded from 10× Genomics, version 2020-April). Expression matrices and spatial coordinate files generated by Space Ranger are used for the next analysis.

### 2.4 Normalization, integration, and clustering of spatial transcriptomic data

We used Seurat (Hao et al., 2021) (version 4.3.0) to perform normalization, integration, and clustering. Expression matrices are read into the R environment, and a Seurat object is created for each sample. The expression count matrices are normalized using the Seurat function SCTransform with the parameter variable.features.n = 9,000, while leaving other parameters as default. Subsequently, we used the Seurat function FindClusters to cluster each sample and annotate them with H&E images. These four Seurat objects are integrated using the Seurat functions SelectIntegrationFeatures, PrepSCTIntegration, FindIntegrationAnchors, and IntegrateData.

For the function SelectIntegrationFeatures, we use the customized parameter nfeatures = 9,000. In FindIntegrationAnchors, we set customized parameters normalization. Method = “SCT,” reduction = “rpca,” k.anchor = 4, and dims = 1:30. Similarly, in IntegrateData, we set customized parameters normalization. Method = “SCT” and dims = 1:50. Other parameters are set as default. Finally, the function RunTSNE is used to perform dimension reduction.

### 2.5 Cell type identification by RCTD

We utilized RCTD (Cable et al., 2022) for cell type identification. Single-cell data are obtained from the Broad Institute with accession code SCP503 (Richards et al., 2021). Raw count data are input into RCTD and run with the parameter doublet_mode set to “doublet.”

### 2.6 Differentially expressed genes and enrichment analysis

**Figure 1:**
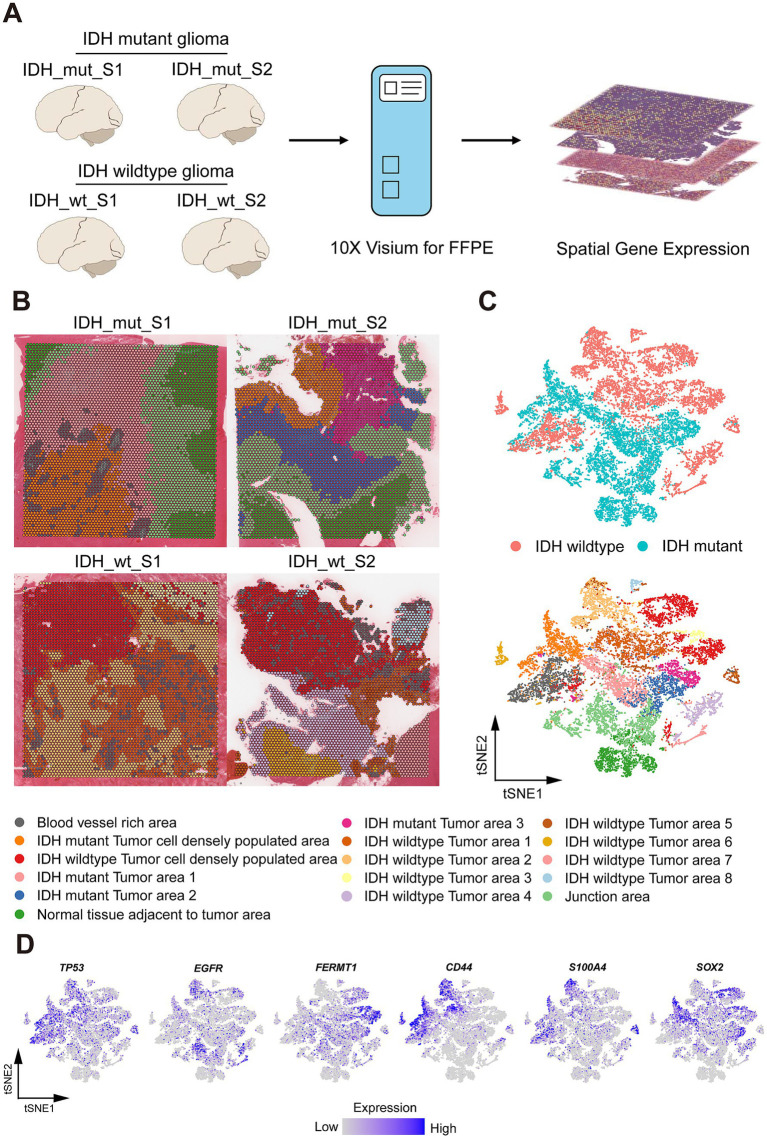
Spatial Profile of Glio-IDH-wt and Glio-IDH-mut. (A) Workflow of glioblastoma sample collection, spatial transcriptomics experiment, and data analysis; (B) Spatial cluster identities of 4 glioblastoma samples, colored by spatial region (bottom); (C) tSNE plot of 4 glioblastoma samples, colored by spatial region (bottom) and Glio-IDH-wt and Glio-IDH-mut, respectively. Each point indicates one spot of spatial transcriptomics; (D) Expression pattern of glioblastoma marker genes (*TP53*, *EGFR*, and *FERMT1*) and glioblastoma stem cell (GSC) markers (*CD44, S100A4*, and *SOX2*).

To analyze the expression of DEGs between the tumor area and normal tissue adjacent to the tumor area (NAT) of Glio-IDH-wt and Glio-IDH-mut, we use the Seurat function FindMarkers to perform the Wilcox rank sum test between Glio-IDH-wt tumor cell densely populated area and Glio-IDH-mut tumor cell densely populated area against NAT. Genes with avg_log_2_FC > 1, p_val_adj < 0.05 and genes with avg_log2FC < −1, p_val_adj < 0.05 are used to perform Gene Ontology (GO) and Kyoto Encyclopedia of Genes and Genomes (KEGG) enrichment analysis with R package clusterProfiler (Wu et al., 2021) (version 4.8.3) with function enrichGO and enrichKEGG. For Venn diagram showed in Figure 1B, we choose differentially expressed genes with p_val_adj < 0.05, avg_log2FC > 1 and pct.1 > pct.2. In our dataset, genes with significantly higher expression in tumor cell densely populated area compared to NAT were selected, as well as the increased level of genes in tumor tissues compared with normal tissues in the online database (GEPIA) (Tang et al., 2019). Meanwhile, the correlation between DEGs and overall survival were analyzed using GEPIA database. For differential expression analysis between Glio-IDH-wt and Glio-IDH-mut tumor area, we first remove spots from Normal tissue adjacent to the tumor area, blood vessel rich area, and junction area. Then Seurat function FindMarkers with parameter min.pct > 0.3 is used for analysis. Genes with avg_log2FC > 0.5, p_val_adj < 0.05, pct.1 > 0.3 and pct.2 > 0.3 are used for GO enrichment analysis. Gene Expression Profiling Interactive Analysis 2 (GEPIA2) data are downloaded and illustrated with the Python package GEPIA (version 0.3).

### 2.7 Module score analysis

For the gene module score, we use the Seurat function AddModuleScore to calculate. The ubiquitin gene list is obtained from iUUCD (Zhou et al., 2018).^1^ The kinase gene list is downloaded from KLIFS (Kanev et al., 2021).^2^ RNA binding protein gene lists are downloaded from previous studies (Wang et al., 2020). All gene lists used in this study are uploaded as Supplementary material.

## 3 Results

### 3.1 Spatial distribution profile of Glio-IDH-wt and Glio-IDH-mut

**Figure 2:**
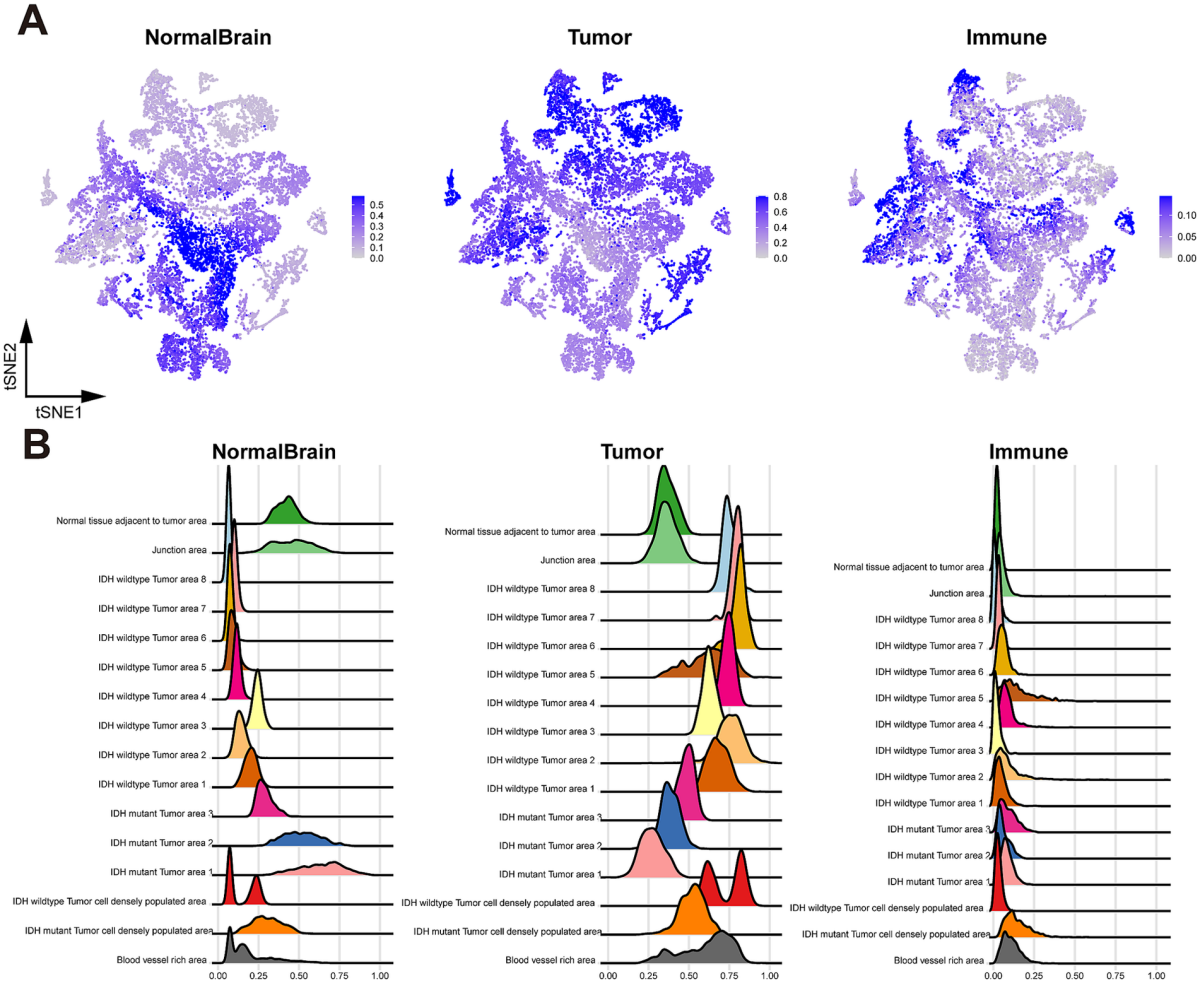
Cell type identification with RCTD. (A) tSNE plot colored by cell type weights of normal brain cells (left), tumor cells (middle), and immune cells (right); (B) Ridge plot of cell type weights of normal brain cells (left), tumor cells (middle), and immune cells (right), grouped by spatial region.

The spatial distribution profile of Glio-IDH-wt (n = 2) and Glio-IDH-mut (n = 2) was analyzed using a structured workflow (Figure 2A). Each section’s spatial clustering was determined using hematoxylin and eosin staining and cell markers, with four samples categorized into 4 distinct clusters through t-distributed stochastic neighbor embedding (tSNE) plots (Figures 2B,C). These clusters included blood vessel-rich area, tumor cell densely populated area, normal tissue adjacent to tumor area, and junction area. Additionally, biomarkers associated with glioma progression (*TP53*, *EGFR*, and *FERMT1*) and glioma stem cells (*CD44*, *S100A4*, and *SOX2*) were examined across different spatial regions of both Glio-IDH-wt and Glio-IDH-mut (Figure 2D). Specifically, *TP53*, *CD44*, and *S100A4* were mainly enriched in the tumor area and blood vessel-rich area of Glio-IDH-wt, and tumor cell densely populated area of Glio-IDH-mut, *EGFR* was mainly enriched in tumor area of Glio-IDH-wt and junction area of Glio-IDH-mut, *FERMT1* was mainly enriched in tumor cell densely populated area of Glio-IDH-wt and tumor area of Glio-IDH-mut, *SOX2* was mainly enriched in tumor cell densely populated area of Glio-IDH-wt and tumor cell densely populated area and tumor area of Glio-IDH-mut, while these genes were not detected in normal tissue adjacent to tumor area of Glio-IDH-mut. In summary, the spatially defined region of four diffuse high-grade glioma samples is reasonable and reliable.

### 3.2 Cell type analysis of spatially defined regions

**Figure 3:**
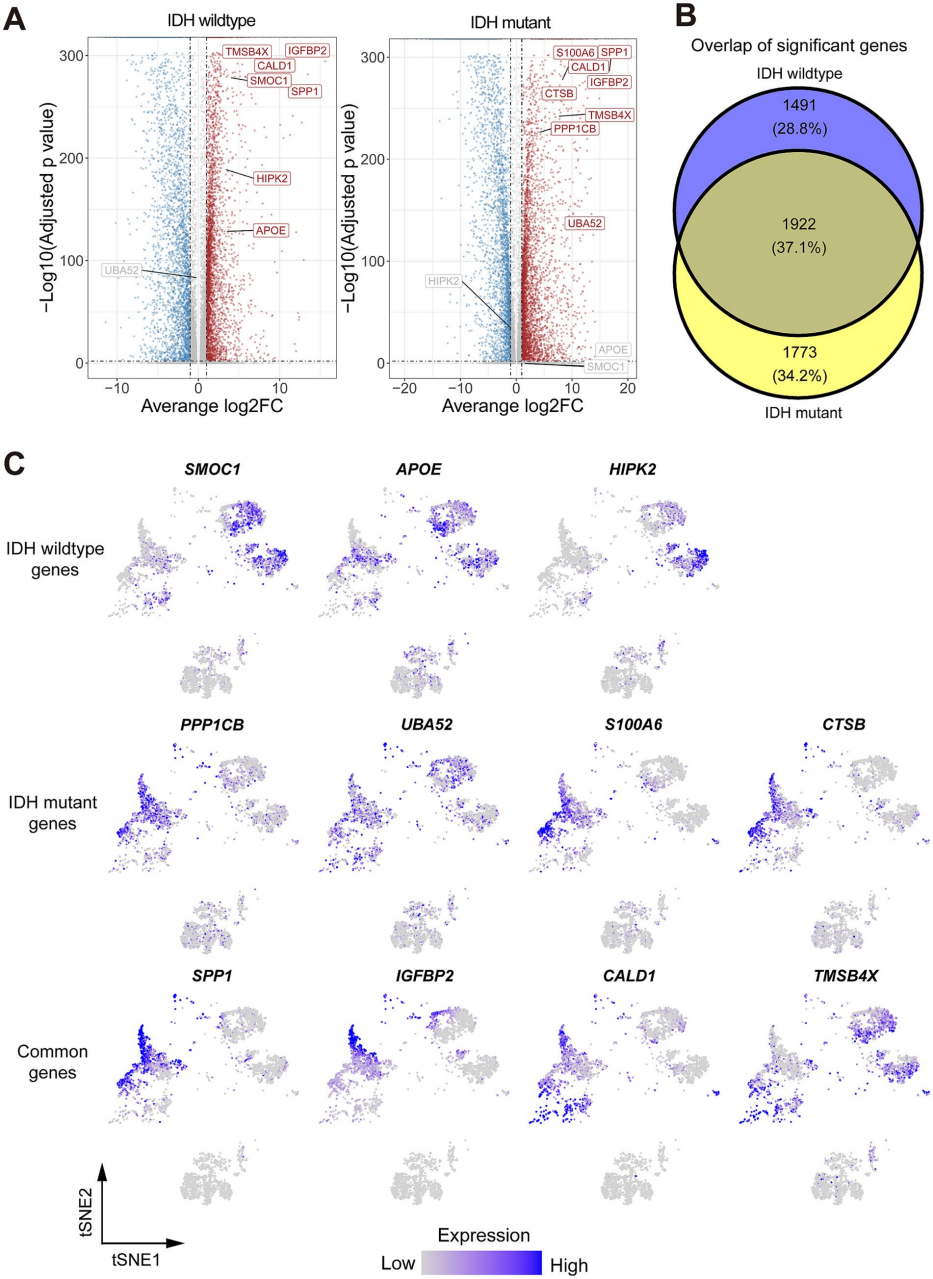
Identification of differentially expressed genes (DEGs) and their related pathways in both Glio-IDH-wt and Glio-IDH-mut. (A) Volcano plot showing DEGs between tumor area and normal tissue adjacent to the tumor area. Genes with average log2(Fold change) > 1 and adjusted *p*-value < 0.01 have higher expression levels in the tumor area and are colored with red in the volcano plot. Genes with average log2(Fold change) < −1 and adjusted *p*-value < 0.01 have higher expression levels in normal tissue adjacent to the tumor area and colored with blue in the volcano plot; (B) A Venn diagram of upregulated DEGs in tumor tissues of both Glio-IDH-wt and Glio-IDH-mut; (C) tSNE plot of upregulated DEGs in spatially defined region of both Glio-IDH-wt and Glio-IDH-mut.

To enhance the characterization of tissue heterogeneity in both Glio-IDH-wt and Glio-IDH-mut, we performed a sub-clustering analysis of cell type across various spatial regions. As depicted in Figure 3, normal brain cells were primarily found in the normal tissue adjacent to the tumor area and junction area of Glio-IDH-mut, glioma cells were distributed in the tumor cell densely populated area, blood vessel-rich area, and tumor area of both Glio-IDH-wt and Glio-IDH-mut, as well as the percentage of immune cells in the tumor cell densely populated area and tumor area of Glio-IDH-mut were higher than those in the Glio-IDH-wt.

### 3.3 Differentially expressed gene analysis of Glio-IDH-wt and Glio-IDH-mut

To address the lack of effective therapeutic targets for diffuse high-grade glioma, we identified the expression profiles of DEGs in Glio-IDH-wt and Glio-IDH-mut using the Seurat function FindMarkers and clustering package. Our analysis results showed that 6,759 DEGs (3,648 upregulated and 3,111 downregulated) in the cancerous tissues of Glio-IDH-wt (Supplementary Table S1). Conversely, a total of 7,435 DEGs (4,113 upregulated and 3,322 downregulated) were identified in the cancerous tissues of Glio-IDH-mut compared to adjacent normal tissues (Figure 1A; Supplementary Table S2). Venn diagrams identified a total of 1,922 upregulated DEGs in spatially defined tumor regions of both Glio-IDH-wt and Glio-IDH-mut (Figure 1B). Meanwhile, a total of 1,491 DEGs were only enhanced in the umor regions of Glio-IDH-wt and 1,773 upregulated DEGs in tumor regions of Glio-IDH-mut. Additionally, tSNE plots showed that the upregulated DEGs were mainly distributed in the spatially defined tumor cell densely populated area of both Glio-IDH-wt and Glio-IDH-mut (Figure 1C). Moreover, the top 3 upregulated DEGs (*SMOC1*, *APOE*, and *HIPK2*) of Glio-IDH-wt and the top 4 upregulated DEGs of Glio-IDH-mut (*PPP1CB*, *UBA52*, *S100A6*, and *CTSB*) and both Glio-IDH-wt and Glio-IDH-mut (*SPP1*, *IGFBP2*, *CALD1*, and *TMSB4X*) in glioblastoma multiforme (GBM) and brain lower grade glioma (LGG) were evaluated using the GEPIA database (Supplementary Figure S1), and these upregulated DEGs showed significant correlations with overall survival (Supplementary Figure S2).

### 3.4 Biological pathway enrichment analysis of upregulated DEGs in Glio-IDH-wt and Glio-IDH-mut

**Figure 4:**
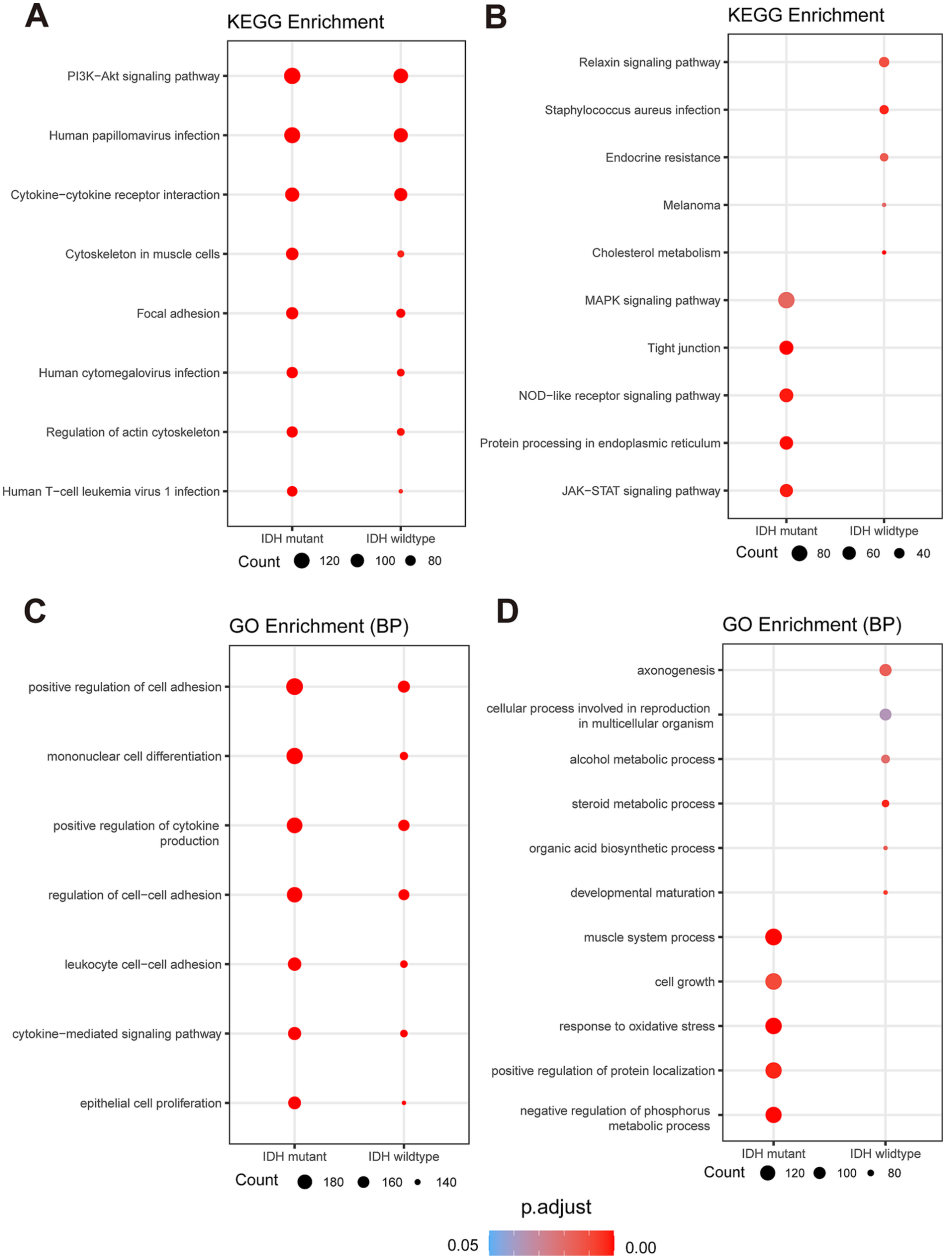
Spatial and functional characterization of DEGs in Glio-IDH-wt and Glio-IDH-mut. Dot plot of enriched KEGG (A,B) and GO (C,D) terms for upregulated DEGs in tumor area of Glio-IDH-wt and Glio-IDH-mut. Genes with avg_log2FC > 1, p_val_adj < 0.05 and genes with avg_log2FC < −1, p_val_adj < 0.05 are used to perform GO and KEGG enrichment analysis.

Based on the above results, the main functional pathways and biological mechanisms by which DEGs play a critical role in the onset and progression of diffuse high-grade gliomas are the next major thing we urgently need to understand, so we performed KEGG analysis and GO analysis on this basis. KEGG enrichment analysis revealed that the upregulated DEGs in tumor areas of both Glio-IDH-wt and Glio-IDH-mut were mainly enriched in eight pathways, including PI3K/Akt signaling pathway, human papillomavirus infection, cytokine-cytokine receptor interaction, cytoskeleton in muscle cells, focal adhesion, human cytomegalovirus infection, regulation of actin cytoskeleton, and human T-cell leukemia virus 1 infection (Figure 4A). Meanwhile, the upregulated DEGs in tumor areas of Glio-IDH-mut were enriched in five pathways, including MAPK signaling pathway, tight junction, NOD-like receptor signaling pathway, protein processing in endoplasmic reticulum, and JAK–STAT signaling pathway, while five pathways were enriched in tumor areas of Glio-IDH-wt such as relaxin signaling pathway, *staphylococcus aureus* infection, endocrine resistance, melanoma, and cholesterol metabolism (Figure 4B). Additionally, GO enrichment analysis (biological process) revealed that these upregulated DEGs in both Glio-IDH-wt and Glio-IDH-mut were mainly enriched in pathways such as positive regulation of cell adhesion, mononuclear cell differentiation, positive regulation of cytokine production, regulation of cell–cell adhesion, leukocyte cell–cell adhesion, cytokine-mediated signaling pathway, and epithelial cell proliferation (Figure 4C). For Glio-IDH-mut, the upregulated DEGs were enriched in biological pathways such as muscle system process, cell growth, response to oxidative stress, positive regulation of protein localization, and negative regulation of phosphorus metabolic process (Figure 4D). For Glio-IDH-wt, the upregulated DEGs were enriched in biological pathways including axonogenesis, cellular process involved in reproduction in multicellular organism, alcohol metabolic process, steroid metabolic process, organic acid biosynthetic process, and developmental maturation (Figure 4D).

### 3.5 Functional role of ubiquitination, RNA binding protein, and kinase in the progression of Glio-IDH-wt and Glio-IDH-mut

**Figure 5:**
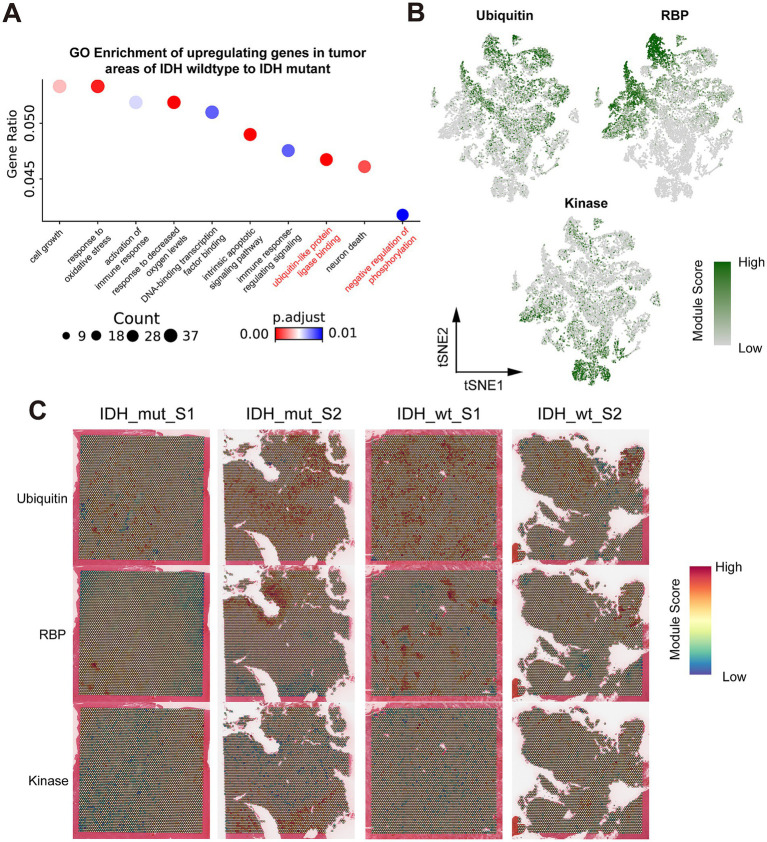
Spatial transcriptomics resolves the key regulatory genes in ubiquitination, RNA binding protein, and kinase in Glio-IDH-wt and Glio-IDH-mut. (A) GO enrichment of genes which upregulating in IDH wildtype tumor areas, compared to IDH mutant tumor areas; (B) tSNE plot colored by the gene module ubiquitin, RBP, and kinase, grouped by spatial region; (C) Spatial map of gene module scores for ubiquitin, RBP, and kinases.

Protein post-translational modification, such as ubiquitination and phosphorylation, constitute essential molecular mechanisms influencing the malignant behaviors of cancer cells and are crucial in various tumor developments (Pan and Chen, 2022). Meanwhile, the advancement of proteomics and epigenetics has identified numerous RNA-binding proteins, offering potential therapeutic targets for glioma (Liu et al., 2024). As expected, GO enrichment analysis illustrated the upregulated DEGs in tumor area of Glio-IDH-wt were mainly enriched in cell growth, response to oxidative stress, activation of immune response, response to decreased oxygen levels, DNA-binding transcription factor binding, intrinsic apoptotic signaling pathway, immune response-regulating signaling, ubiquitin-like protein ligase binding, and negative regulation of phosphorylation compared with the Glio-IDH-mut group (Figure 5A; Supplementary Table S3). Combining the current research hotspots and our research interests, we decided to take the ubiquitination, RNA binding protein, and kinase as the emphasis of subsequent research. Correspondingly, tSNE plots illustrated heightened expression of key regulatory genes in ubiquitination and RNA binding proteins within spatially defined tumor regions of both Glio-IDH-wt and Glio-IDH-mut (Figures 5B,C) Conversely, the expression of key regulatory genes in kinase was enhanced in the normal tissue adjacent to the glioma tumor. Moreover, the gene lists associated with ubiquitination, RNA binding protein, and kinase were shown in Supplementary Table S4. These findings suggested that aberrant protein post-translational modification and dysregulation of RNA-binding proteins play an important role in the progression of diffuse high-grade gliomas.

## 4 Discussion

High-throughput sequencing of cancer patient gene expression can significantly enhance personalized and precise treatment strategies for clinicians (Morganti et al., 2019). Most current studies primarily focus on identifying overall gene expression differences between cancerous tissues and adjacent non-tumor tissues, lacking spatial information on complex tissue structures that could elucidate spatial specificity in gene expression (Marx, 2021). The landscape of tumor research has been transformed by advancements in sequencing techniques, yet conventional methods fail to integrate gene expression data with spatial cellular information. In contrast, spatial transcriptome sequencing, an emerging sequencing technique distinct from RNA-sequencing (RNA-seq) and single-cell RNA-seq (scRNA-seq), provides spatial context to gene expression data, offering insights into cellular positional relationship with tissue sections (Burgess, 2019; Wagner et al., 2019). In this study, spatial transcriptomic technology was employed to explore spatial heterogeneity in diffuse high-grade gliomas, characterizing their spatial features and comparing key DEGs between Glio-IDH-mut and Glio-IDH-wt, as well as within the tumors.

Our study identified 5,677 upregulated and 5,016 downregulated DEGs between tumor and non-tumor tissues of diffuse high-grade gliomas, highlighting notable upregulated genes such as *SMOC1*, *APOE*, *HIPK2*, *PPP1CB*, *UBA52*, *S100A6*, *CTSB*, *SPP1*, *IGFBP2*, *CALD1*, and *TMSB4X*. Previous studies have validated that these upregulated DEGs, except for *SMOC1* and *UBA52*, were associated with poor prognosis in malignant tumors and presented limited treatment options (Zhang et al., 2014; Abdulla et al., 2017; An et al., 2021; Hu et al., 2021; Liu et al., 2021; Lin et al., 2022; Conte et al., 2023; Zhang Z. et al., 2024; Zhang X. et al., 2024). In gliomas, *SMOC1* (Wang J. et al., 2021), *S100A6* (Hong et al., 2023), *CTSB* (Ma et al., 2022), *SPP1* (Chen et al., 2019), and *IGFBP2* (Moore et al., 2009) serve as a potential therapeutic target in glioma. Similarly, *APOE*-mediated lipid transport may represent a new therapeutic target in brain tumors (Nicoll et al., 2003). Sardina et al. (2023) summarized that *HIPK2* may serve as a therapeutic target and diagnostic or prognostic marker in neurological disorders. *PPP1CB* has been shown to serve as an oncogene in high-grade glioma by activating the Ras-ERK, JAK3-STAT3, and PI3K-Akt pathways (Chiarle et al., 2008; Aghajan et al., 2016). *UBA52*, one of the genes associated with ubiquitin, encodes a fusion protein consisting of ubiquitin at the N-terminus and ribosomal protein L40 at the C-terminus (Dubois et al., 2020; Cockram et al., 2021), which has been reported to contribute to the development and progression of various tumors, including colorectal cancer (Zhou et al., 2019) and non-small cell lung cancer (Wang et al., 2019). Other studies have demonstrated that *S100A6* (Qi et al., 2023), *CTSB* (Zhang et al., 2020), and *CALD1* (Ma et al., 2024) have been proven to promote cancer cell malignant phenotypes, such as proliferation, migration, invasion, and epithelial-mesenchymal transition. Numerous studies have proved that *SPP1*-positive macrophages act as metastasis accelerators that promote tumor progression (Liu et al., 2023; Du et al., 2024). A recent study showed that cancer-associated fibroblast-associated gene *IGFBP2* facilitated glioma progression by activation of M2 macrophage polarization (Zhang X. et al., 2024). However, the functional role of *SMOC1*, *APOE*, *HIPK2*, *UBA52*, *S100A6*, *CTSB*, *SPP1*, *IGFBP2*, *CALD1*, and *TMSB4X* in the onset and progression of diffuse high-grade glioma remains unclear.

Exiting studies have linked aberrant activation of the PI3K/Akt pathway (Barzegar Behrooz et al., 2022), human papillomavirus infection (Limam et al., 2020), cytokine-cytokine receptor interaction (Wang J. J. et al., 2021), and human T-cell leukemia virus 1 infection (Jin et al., 2006) with glioma development and progression. In our analysis, we observed similar results, where upregulated DEGs in tumor regions of both Glio-IDH-wt and Glio-IDH-mut exhibited enrichment in pathways such as PI3K/Akt, human papillomavirus infection, cytokine-cytokine receptor interaction, human T-cell leukemia virus 1 infection, cytoskeleton in muscle cells, focal adhesion, human cytomegalovirus infection, regulation of actin cytoskeleton, and human T-cell leukemia virus 1 infection. Meanwhile, the upregulated DEGs of Glio-IDH-mut were mainly enriched in “MAPK signaling pathway,” “tight junction,” “NOD-like receptor signaling pathway,” “protein processing in endoplasmic reticulum,” and “JAK–STAT signaling pathway.” Previous studies have proved that activation of the MAPK pathway (Pan et al., 2023), endoplasmic reticulum stress (Markouli et al., 2020), NOD-like receptor pathway (Chen et al., 2024), and JAK–STAT pathway (Zhang et al., 2019) accelerated glioma progression. Our results also showed that the enrichment analysis results of upregulated DEGs of Glio-IDH-wt were mainly enriched in the relaxin signaling pathway, *staphylococcus aureus* infection, endocrine resistance, melanoma, and cholesterol metabolism. Several studies have proved that the relaxin pathway may serve as a potential therapeutic approach to control malignant tumors (Neschadim et al., 2015; Klonisch et al., 2017). Cuervo et al. (2010) found that *staphylococcus aureus* infection may increase mortality in cancer patients. Wang T. et al. (2023), reported that cholesterol transport served as a novel potential target pathway for IDH-mut glioma. Moreover, GO analysis revealed that the upregulated DEGs of both Glio-IDH-wt and Glio-IDH-mut were related to positive regulation of cell adhesion, mononuclear cell differentiation, positive regulation of cytokine production, regulation of cell–cell adhesion, epithelial cell proliferation, and so on, which is consistent with the former studies (Wu et al., 2019). Furthermore, GO analysis found that axonogenesis, cellular process involved in reproduction in multicellular organism, alcohol metabolic process, steroid metabolic process, organic acid biosynthetic process, and developmental maturation were enriched in Glio-IDH-wt, whereas Glio-IDH-mut showed enrichments of muscle system process, cell growth, response to oxidative stress, positive regulation of protein localization, and negative regulation of phosphorus metabolic process. The above results indicated that these DEGs play a crucial role in the development and progression of diffuse high-grade gliomas.

Post-translational modification of proteins is a crucial regulatory mechanism that alters their physical and chemical properties, conformation, and binding ability of proteins, thus affecting their activity, stability, and function (Su et al., 2017). Research on protein post-translational modification in malignant tumors, such as glioma, currently emphasizes on phosphorylation, ubiquitination, glycosylation, and acetylation. These modifications regulate protein function, modulate signaling pathways, and impact downstream gene expression, affecting cancer cell proliferation, invasion, apoptosis, drug resistance, and chemotherapy sensitivity (Han et al., 2018; Cao and Yan, 2020). Moreover, protein post-translational modification is a significant focus in epigenetic research, and recent studies highlight its relevance to glioma pathogenesis, offering diagnostic and therapeutic targets (Pienkowski et al., 2023). Our study reveals that key regulatory genes involved in ubiquitination and RNA binding protein were highly expressed in spatially defined tumor regions of both Glio-IDH-wt and Glio-IDH-mut, while key regulatory genes in kinase were predominantly enhanced in adjacent normal tissue. We identified the top 6 key regulatory genes in ubiquitination (*TRIM54*, *PPARG*, *KLHL35*, *DNAI1*, *WDR78*, and *CORO1A*), RNA binding protein (*ENO2*, *LGls3*, *HSP90AA1*, *HSPA1B*, and *HK2*), and kinase (*KALRN*, *LRRK1*, *MET*, *PDK1*, *PET*, and *TRIB3*). Notably, these genes, such as *PPARG*, *HK2*, and *PDK1*, play critical roles in the regulation of aerobic glycolysis in cancer cells, which is associated with the IDH phenotype in GBM cells (Strickland and Stoll, 2017; Dong et al., 2023; Venneti et al., 2023).

In conclusion, our systematic investigation of spatially distributed DEGs in diffuse high-grade gliomas identified 10 key genes and their associated signaling pathways. These findings are supported by existing literature and databases linking these differential genes to malignant tumor progression. This study contributes to the development of personalized treatment strategies for diffuse high-grade gliomas. However, the functional role and underlying mechanism of SMOC1, HIPK2, UBA52, S100A6, PPP1CB, CALD1, and TMSB4X in the occurrence and development of diffuse high-grade gliomas was still unknown. This is also the main point of our future research.

## Data Availability

All data generated in this work are uploaded to the National Genomics Data Center (https://ngdc.cncb.ac.cn) with the BioProject accession number PRJCA027587. All code used in this work is uploaded to GitHub (https://github.com/fuyu-sama/gbm).

## Footnotes

^1^ http://iuucd.biocuckoo.org/

^2^ https://klifs.net/

## Supplementary material

SUPPLEMENTARY FIGURE S1 Expression of *SMOC1*, *APOE*, *HIPK2*, *PPP1CB*, *UBA52*, *S100A6*, *CTSB*, *SPP1*, *IGFBP2*, *CALD1*, and *TMSB4X* in glioblastoma multiforme (GBM) and brain lower grade glioma (LGG) was examined by GEPIA database.

SUPPLEMENTARY FIGURE S2 Correlation of the expression of *SMOC1*, *APOE*, *HIPK2*, *PPP1CB*, *UBA52*, *S100A6*, *CTSB*, *SPP1*, *IGFBP2*, *CALD1*, and *TMSB4X* with overall survival of patient with glioblastoma multiforme (GBM) and brain lower grade glioma (LGG) was examined by GEPIA database.

SUPPLEMENTARY TABLE S1 DEGs were identified in the cancerous tissues of Glio-IDH-wt compared to adjacent normal tissues.

SUPPLEMENTARY TABLE S2 DEGs were identified in the cancerous tissues of Glio-IDH-mut compared to adjacent normal tissues.

SUPPLEMENTARY TABLE S3 Gene list associated with upregulated DEGs in tumor area of Glio-IDH-wt compared with Glio-IDH-mut.

SUPPLEMENTARY TABLE S4 Gene list associated with ubiquitination, RNA binding protein, and kinase.
