# Supplementary figures and images for: Spatial transcriptomics analysis identifies therapeutic targets in diffuse high-grade gliomas
Source: Front Mol Neurosci. 2024 Oct 24;17:1466302. doi: 10.3389/fnmol.2024.1466302 (PMC11552449; doi:10.3389/fnmol.2024.1466302)

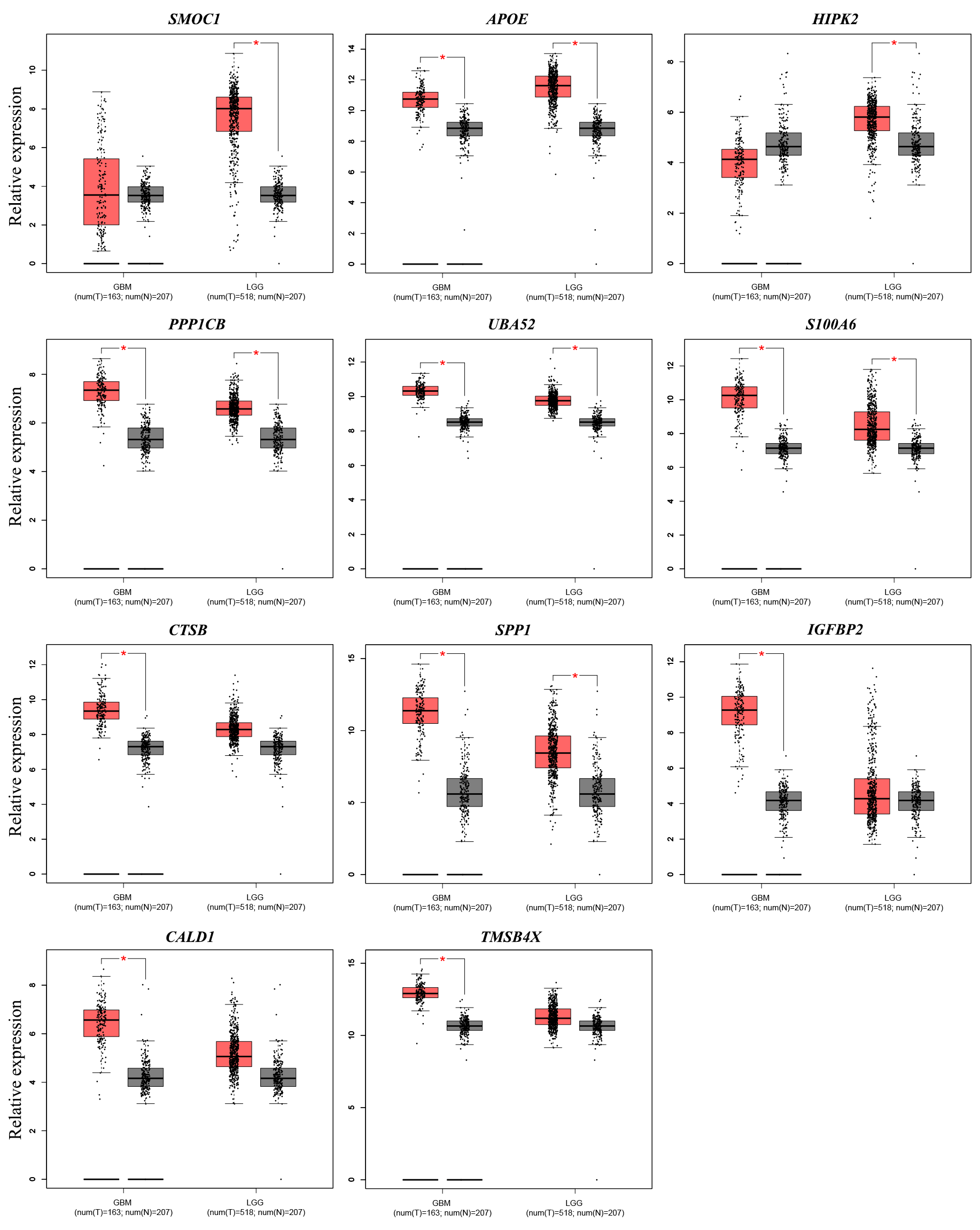

Supplement: SUPPLEMENTARY FIGURE S1 — Expression of SMOC1, APOE, HIPK2, PPP1CB, UBA52, S100A6, CTSB, SPP1, IGFBP2, CALD1, and TMSB4X in glioblastoma multiforme (GBM) and brain lower grade glioma (LGG) was examined by GEPIA database. [file Image_1.TIF]

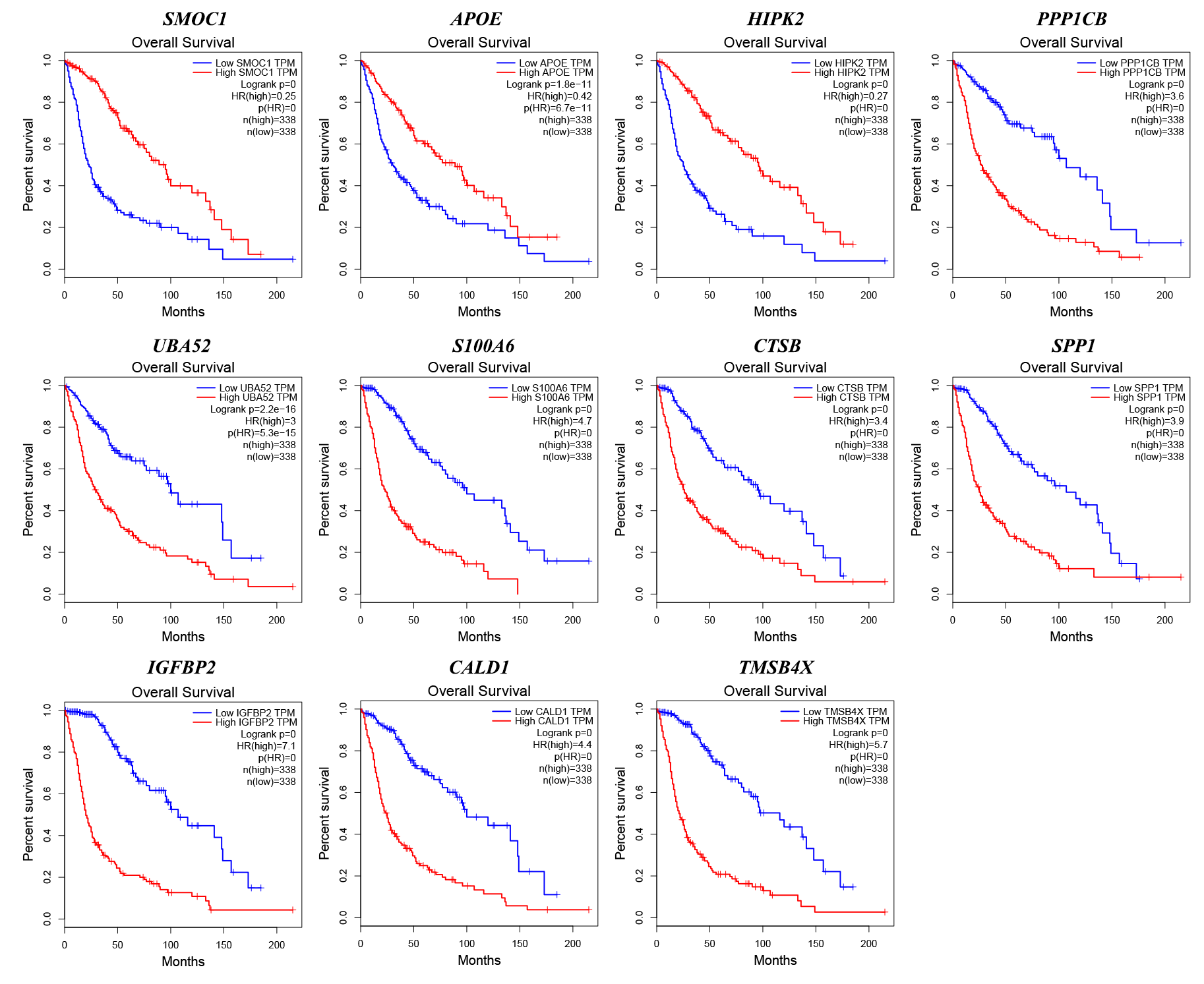

Supplement: SUPPLEMENTARY FIGURE S2 — Correlation of the expression of SMOC1, APOE, HIPK2, PPP1CB, UBA52, S100A6, CTSB, SPP1, IGFBP2, CALD1, and TMSB4X with overall survival of patient with glioblastoma multiforme (GBM) and brain lower grade glioma (LGG) was examined by GEPIA database. [file Image_2.TIF]
